# Enhancing Saltiness Perception Using Chitin Nanomaterials

**DOI:** 10.3390/polym11040719

**Published:** 2019-04-19

**Authors:** Wan-Chen Tsai, Shang-Ta Wang, Ke-Liang Bruce Chang, Min-Lang Tsai

**Affiliations:** Department of Food Science, National Taiwan Ocean University, 2, Pei-Ning Road, Keelung 20224, Taiwan; f22925661@gmail.com (W.-C.T.); awang@hughesbiotech.com (S.-T.W.); klchang@mail.ntou.edu.tw (K.-L.B.C.)

**Keywords:** chitin, nanofiber, nanocrystal, saltiness perception, sodium reduction

## Abstract

In the present study, we prepared and characterized chitin nanomaterials with different diameters, lengths, and degree of deacetylation (DD), and investigated their capability for enhancing saltiness perception. Chitin was isolated from squid pens and transformed into chitin nanofiber (CNF), deacetylated chitin nanofiber (DACNF), and chitin nanocrystal (CNC) by ultrasonication, alkali treatment followed by ultrasonication and acid hydrolysis, respectively. The diameters of CNF, CNC and DACNF were 17.24 nm, 16.05 nm and 15.01 nm while the lengths were 1725.05 nm, 116.91 nm, and 1806.60 nm, respectively. The aspect ratios of CNF and DACNF were much higher than that of CNC. The crystalline indices of CNF and CNC were lower than that of original β-chitin, suggesting that ultrasonication and acid hydrolysis might change the molecular arrangement in crystalline region of chitin. The zeta-potentials were between 19.73 nV and 30.08 mV of chitin nanomaterials in distilled water. Concentrations of chitin nanomaterials (40–74 μg/mL) showed minimal effect on zeta-potential, whereas increasing the level of NaCl reduced the zeta-potential of solution. Moreover, NaCl solution (0.3%) with chitin nanomaterials addition produced significant higher saltiness perception than that of solution with NaCl alone. Therefore, chitin nanomaterials may be promising saltiness enhancers in the food industry.

## 1. Introduction

Sodium chloride (NaCl), a ubiquitous sodium salt, is commonly used to season food. As a food additive, NaCl not only produces the saltiness perception, but also lowers the water activity of foods. Consequently, salt can inhibit the growth of microbial and prolongs the shelf life of foods. However, excess sodium-intake leads to increased risk of non-communicable diseases (NCDs) including stroke, hypertension, and cardiovascular disease, which are the leading causes of death globally [1]. The World Health Organization (WHO) (2007) recommended a daily NaCl intake (RDI) of less than 5 g. Nevertheless, the average daily NaCl intake in the United States, United Kingdom and Asia is approximately 8.2–9.4 g, 9.4 g, and 12.0 g, respectively, which are much higher than the RDI suggested by WHO [2]. Hence, the techniques to reduce the salt levels in foods should be considered in order to help bring dietary sodium-intake closer to those recommended for promoting public health. Many countries have undertaken policies to deal with this issue. For example, in Finland, the labeling of salt has been determined by national regulations. Categories of “reduced salt” and “heavily salted” in different foods were itemized in national standards to help consumers choose food with less salt [3]. However, a lower sodium content is often associated with a lower consumer acceptance, which may reduce certain products’ market share as consumers no longer enjoy the flavor of products with reduced salt [4]. Moreover, research indicated that 75–80% of sodium intakes are from processed food products. Therefore, it is necessary for the food industry to bring strategies for enhancing consumers’ perception of food saltiness into practice as well [5,6].

Common methods for reducing sodium level and promoting saltiness perception of food products include using substitutes such as KCl, CaCl_2_, and MgSO_4_ [7], as well as adding flavor enhancers such as citric acid and monosodium glutamate [8]. However, those metal salts and acids have astringency and metallic flavor, their actual incorporations in food preparations are therefore limited. In addition, the rate of release of sodium from the food matrix and the dissolution of salts from dry products have been proved crucial for saltiness perception of foods. As a consequence, approaches such as modulating the shapes and sizes of salt crystal, and altering the texture of food matrix have been recently explored for their potentials as salt reduction alternatives [9,10]. Saltiness is primarily created by free sodium ions rather than the bounded ones. When dry foods are consumed, 70 to 95% of sodium (or salt) may remain in the food matrix as bounded form after swallowing, which indicates that a significant proportion of sodium might be swallowed without being perceived [11]. Accordingly, the more sodium ions released from food matrix and dissolved, the less salt had to be added in foods and less sodium would be consumed [6]. On the other hand, the presence of negatively charged groups in ionic food matrix (ex. milk protein, soy protein, xanthan gum and κ-carrageenan) was shown to reduce salty perception due to increased electrostatic interactions between sodium ions and food matrix [12]. In contrast, positively charged groups provide by food composition may interact with negatively charged groups via electrostatic interactions, which can therefore help release more free sodium ions and induce higher saltiness [13].

Chitin is a nitrogen-containing linear polysaccharide composed of β-1,4 linked units of *N*-acetyl glucosamine, which has positive charge under weak acidic environment. It can be isolated from bio-resources such as shrimp, crab shells and squid pens [13]. Recently, chitin was extensively studied in the biomedical materials field owing to its notable biological properties [14]. Chitin nanomaterials are differed from chitin itself in physical properties. These nanomaterials can easily form biological suspension and overcome the limitation for chitin utilization in food and biomedical fields [15]. Chitin nanofibers (CNFs), 2–5 nm in diameter and about 300 nm in length, is one of the chitin-based novel materials that feature distinct structural, mechanical and optical characteristics against micrometer-scaled fiber materials [16]. It can be produced by grinding [17], ultrasonication [18], microfluidization [19], Star Burst system [20], dynamic high-pressure homogenization [21], and explosive puffing treatment [22] with purified chitin. In addition to CNFs, chitin nanocrystal (CNC) is another chitin nanomaterial that has attracted much attention. CNCs, which can be prepared through acid hydrolysis of chitin, have a fiber or rod-like shape with a lower aspect ratio and a higher crystallinity than CNFs in morphology. They are more easily degradable and poor in elasticity relative to CNFs [19,23,24]. The novel nanomaterials made of chitin can be partially protonated and possess positive surface charges in a pH environment below seven. The positively charges from protonated amino groups in CNF and CNC may interact with the negatively charged ions in food system, such as chloride ions from NaCl and carboxylate ions from proteins. The quantity of free sodium ions in solution may therefore elevate, and this will be followed by increased saltiness [13]. Cured fish fillet were used as a strong model system for testing this hypothesis. Research indicated that fish fillets cured with salt and additional CNF elevated the concentration of free sodium ions in the system, enabling additional free sodium ions in the curing solution to be released into fish fillets [25]. These prior studies suggest that chitin nanomaterials may be promising as a saltiness enhancer in food industry.

It is known that the length and diameter may strongly affect the surface area of chitin nanomaterials. Moreover, the degree of deacetylation (DD) of chitin affect the protonation degree of chitin molecules as well. In this study, we prepared and characterized chitin nanomaterials with various DD, length and diameter. These nanomaterials were introduced into NaCl solutions, in order to examine their effects on the saltiness perception of salt solutions.

## 2. Materials and Methods

### 2.1. Materials

Squid (*Illex argentinus*) pens were donated as a gift from Shin Ho Sing Ocean Enterprise Co., Ltd. (Kaohsiung, Taiwan). Sodium hydroxide, Hydrochloric acid, Potassium bromide were purchased from Sigma-Aldrich Co. (St. Louis, MO, USA). Sodium chloride was purchased from Uniregion bio-tech (Hsinchu, Taiwan).

### 2.2. Preparation of β-Chitin and Deacetylated Chitin

Squid pens were dried, and then ground into an 80–120 mesh size. Each 40 g of powder was immersed in 600 mL of 1 M hydrochloric acid solution for 8 h at room temperature. Subsequently, the powder was washed with 10-fold volume of distilled water 3 times, followed by soaking in 600 mL of 2 M sodium hydroxide for another 8 h at room temperature. It was then soaked in 600 mL of 2 M sodium hydroxide solution at 100 °C for 4 h, then washed until neutral and dried to produce β-chitin [26].

For preparing deacetylated chitin, 30 g of β-chitin powder was added to 750 mL of 33% (*w*/*w*) sodium hydroxide solution. The deacetylation reaction took place at 90 °C for 4 h. The sample was then washed till neutral, then dried and stored at 4 °C until use [27].

### 2.3. Preparation of CNF and Deacetylated Chitin Nanofiber (DACNF)

β-Chitin was used to make 150 mL aqueous chitin suspension at 0.5 g/L. The aqueous suspension was then placed in an ice bath and ultrasonicated at 20 kHz, 500 W by using an ultrasonic processor (ChromTech UP-500, Apple Valley, MN, USA) for 1 and 2 h respectively. The ultrasonication process involved cycles of 9 s of ultrasonication followed by a pause of 4 s. Subsequently, the aqueous solution was centrifuged at 12,000 rpm for 30 min to produce CNF suspensions [13]. DACNF suspension was prepared using deacetylated chitin as material with the same procedures.

### 2.4. Preparation of CNC

β-Chitin was added into 3 N HCl at a ratio of 1:30:50 (chitin:HCl:distilled water, *w*/*v*/*v*), then was centrifuged at 3400 rpm for 15 min. This process was repeated three times. Subsequently, distilled water was added into the precipitant to form suspension, and the suspension was transferred to a dialysis membrane (MWCO = 3500, Orange scientific, OrDial D35, Braine-l’Alleud, Belgium) and dialyzed until neutral. Afterward, appropriate volumes of 1 N HCl solution were used to adjust the final pH of the suspension to 3.0. To impede the possible chitin nanocrystal aggregation, the suspension was introduced to ultrasonic treatment (35 min for the suspension with 5 min intervals). The suspension was subsequently centrifuged at 3000 rpm for 10 min and the supernatant was collected as CNC suspension and stored at 4 °C [28,29].

Finally, CNF, DACNF, or CNC suspensions were lyophilized. The dried samples were weighed and the yields of nanomaterials were determined by using the following formula:

Yield (%) = Sample weight after lyophilization/Weight of chitin material.

### 2.5. Physicochemical Properties

#### 2.5.1. Transmission Electron Microscopy

The morphology of the chitin nanomaterials was observed on TEM (Hitachi, H-7650, Tokyo, Japan). Ten microliters of nanomaterial’s suspension were dropped on copper grids for 5 min, excess was removed by a filter paper. TEM micrographs of chitin nanomaterials were obtained, and were then analyzed by Image J software (Wayne Rasband, National Institutes of Health, Bethesda, MD, USA). The micrograph was divided into four quadrants, and 10–25 objects of nanomaterial were randomly selected from each quadrant to obtain length and diameter of nanomaterials. The aspect ratio (length/diameter) was calculated as well [29].

#### 2.5.2. Fourier-Transform Infrared Spectroscopy

The functional groups and degree of deacetylation (DD) of the chitin and CNFs were determined with Fourier transform infrared spectroscopy (FTIR). The sample powder was mixed with KBr at a ratio of 1:100. The mixture was dried at 60 °C for 3 days to prevent the -OH group from interfering with the FTIR measurements, and was then pressed into pellet form. The absorbance of amide 1 (1655 cm^−1^) and the hydroxyl band (3450 cm^−1^) was measured using an FTIR spectrometer (Bio-Rad FTS-155, Hercules, CA, USA). The hydroxyl group band at 3450 cm^−1^ was utilized as an internal standard for calibrating the disc thickness and adjusting the chitin concentration. Triplicate measurements were averaged and applied to calculate the DD by using the following Equation [13]:

DD (%) = 100 − (A_1655_/A_3450_) × 115 (1)

where A_1655_ and A_3450_ denote the absorbance at 1650 cm^−1^ and 3450 cm^−1^, respectively.

#### 2.5.3. X-ray Diffraction

Crystallinities of chitin nanomaterials were analyzed by X-ray diffractometer (Panalytical B.V, X’Pert Pro MPD, Almelo, The Netherlands). Freeze-dried samples were compressed and mounted on a sample tray. The analysis was carried out with an anode current of 20 mA and an accelerating voltage of 40 kV. Samples were exposed to CuKα radiation at diffraction angles (2θ) from 5° to 40°, and the counting time was 1 s at each angle step (0.1°).

The maximum intensities, I_110_ at 2θ ≈ 20° and I_020_ at 2θ ≈ 10° were measured respectively. The maximum intensity of amorphous diffraction, I_am_, was obtained at 16° as the general baseline [30]. The crystalline indices were calculated using the following formulations:

CrI_110_ = (I_110_ − I_am_)/I_110_ × 100 (2)

CrI_020_ = (I_020_ − I_am_)/I_020_ × 100 (3)

where CrI_110_, and CrI_020_ express the crystalline index at 110 and 020 reflection, respectively.

### 2.6. Properties of Chitin Nanomaterials Suspensions

#### 2.6.1. Zeta Potential

Various quantities of each chitin nanomaterials (CNF, DACNF, and CNC) were dispersed in distilled water to form suspensions with concentrations of 40–74 μg/mL. To investigate the effect of concentration of NaCl on zeta potential, 74 μg/mL of chitin nanomaterials (CNF, DACNF, and CNC) was added into 0.01% and 0.05% NaCl solution for further analysis. Zeta potential analysis of all suspensions was carried out using a dynamic light scatterometer (Zetasizer Nano ZS, Malvern, Worcestershire, UK) [29]. The salt concentrations used in this study were approximated to the maximum of the instrumental limitation of light scatter, which noticed that the sample with excess salt concentration would cause serious electrodes burnout.

#### 2.6.2. Sensory Evaluation

For preparing samples for sensory evaluation, each chitin nanomaterial was used to prepared 80 μg/mL aqueous suspension with a NaCl concentration of 0.3%, respectively. Solutions with 0.3% NaCl as blank control were also obtained. The panel consisted of 30 untrained and volunteered subjects (17 female and 13 male, 21–25 years of age), who were recruited from postgraduates of Department of Food Science, National Taiwan Ocean University (Keelung, Taiwan) to participate in the hedonic sensory tasting. Every panelist was urged to drink taste 10 mL of 0.3% NaCl solution, as well as CNF/NaCl, DACNF/NaCl, and CNC/NaCl suspensions for assessing evaluating the saltiness, which were was evaluated rated using a 7-point scale (1: very weak, 4: moderate, 7: very strong). Before moving on to the next suspension, the panelists were requested to rinse their mouth with water sufficiently to neutralize their taste remove the effect of previous sample on the taste acuity [31].

### 2.7. Statistical Analysis

One-way analysis of variance was used to determine the differences between the experimental groups. If the differences were significant, Tukey’s honest significant difference test was used as the post-hoc test for further analysis. The significance level was set at *p* < 0.05. IBM SPSS Statistics Version 22 software was used for all statistical analyses. 

## 3. Results and Discussion

### 3.1. Yields of Chitin Nanomaterials

The yields of CNF, DACNF and CNC are shown in Table 1. The yield of CNF was much higher than that of DACNF. Generally, the arrangement of β-chitin nanofiber is with parallel structures, and had few hydrogen bonds within molecular chains [32]. Consequently, the yields of CNF and DACNF are assumed to be comparable. However, deacetylated chitin possessed more exposed amino groups than original β-chitin. These exposed groups generated intermolecular forces such as charge interactions between chitin molecules in its nanofibers. As a result, the nanofibers may easily aggregate and precipitate within centrifugation process. These intermolecular interactions may account for the fact that the yield of DACNF is lower than CNF.

The yield of CNC was 44.5% (Table 1). This is comparable to the results from Ma et al. [33] and Pereira et al. [29]. The yields of CNC, prepared through acid hydrolysis, from those previous reports were 40–65%. In addition, ultrasonication method for preparing CNC had slightly higher yield than using acid hydrolysis, but were not statistically different.

### 3.2. TEM Morphology Study

**Figure 1:**
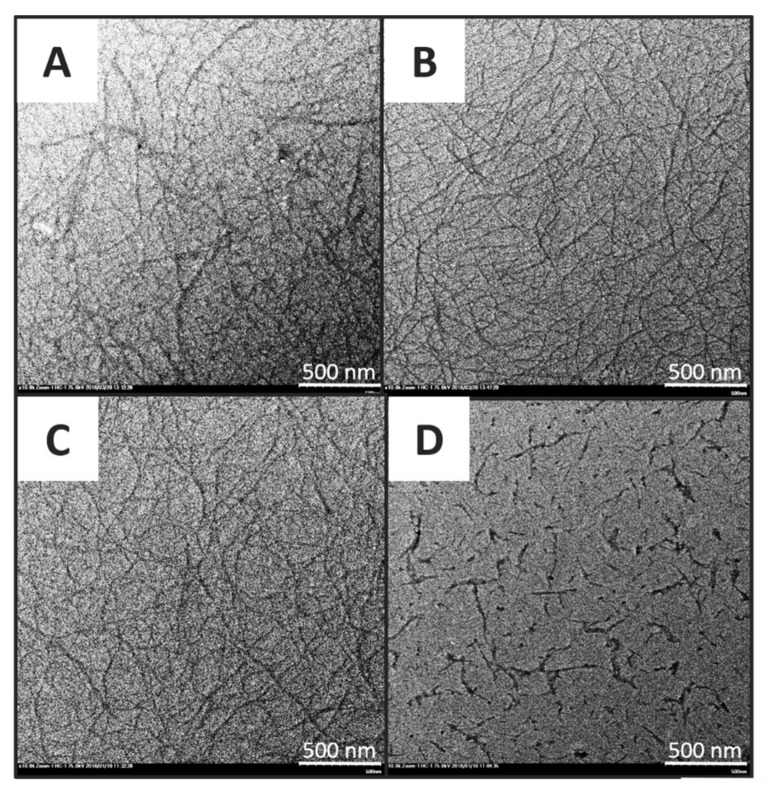
TEM micrograph of (**A**) chitin nanofibers after 1 h ultrasonication; (**B**) after 2 h ultrasonication; (**C**) deacetylated chitin nanofibers; and (**D**) chitin nanocrystals from squid pen.

The morphology of chitin nanomaterials was studied via TEM. Figure 1A showed the micrograph of CNFs produced by 1 h ultrasonication treatment. The fiber exhibited flexible hair-like appearance, and had an average diameter of 17.24 nm. To improve the ultrasonication efficiency, additional 1 h treatment was introduced to the CNFs. The prolonged treatment resulted in CNFs with an average diameter of 15.67 nm, but both CNFs samples were similar in morphology. Liu et al. [18] prepared CNF by using chitin from shrimp shells, underwent 60 kHz, 300 W of ultrasoncation treatment with cycles of 9 s of ultrasonication followed by a pause of 4 s for total treatment times of 5 min and 30 min, respectively. The average diameters of CNFs that they obtained were 105 nm and 20 nm by each treatment. The prolonged time of ultrasonication treatment significantly reduced the average diameter of CNF. In our study, however, no significant reduction in average diameter was observed along with prolonging treatment.

On the other hand, DACNF shared a similar exterior with CNF with an average diameter of 15.01 nm (Figure 1C). CNC, as distinguished with CNFs, exhibited a rod-like appearance with an average diameter of 16.05 nm, and the CNCs were well-dispersed with a few aggregates in the micrograph (Figure 1D).

**Figure 2:**
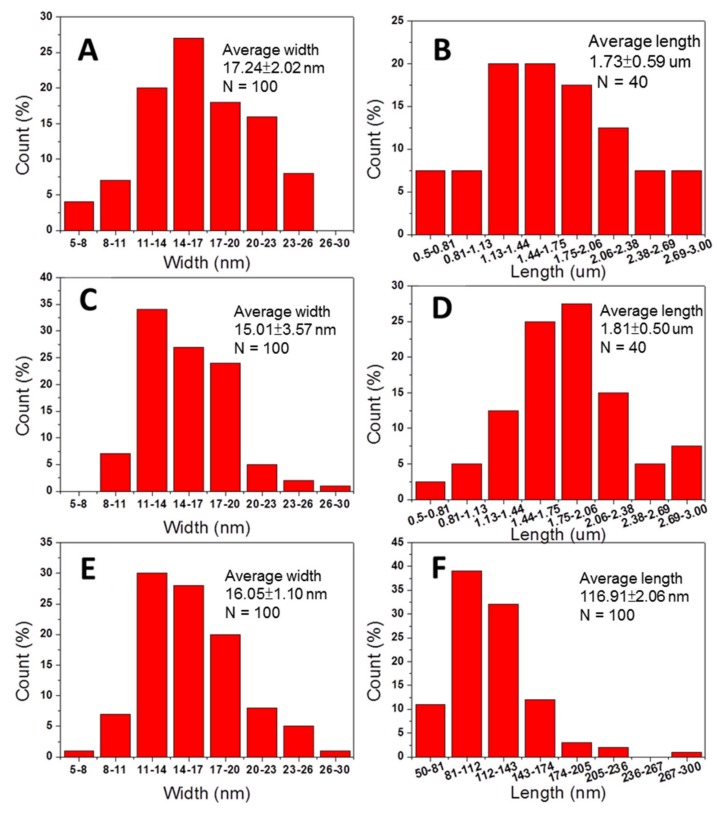
The width and length distribution from TEM of (**A**) width of chitin nanofibers; (**B**) length of chitin nanofibers; (**C**) width of deacetylated chitin nanofibers; (**D**) length of deacetylated chitin nanofibers; (**E**) width of chitin nanocrystals; and (**F**) length of chitin nanocrystals.

Figure 2 showed the diameter and length distribution of each nanomaterial from TEM observation analyzed by Image J software. The average diameters and lengths, and aspect ratios were listed in Table 2. Results indicated that the aspect ratio of CNC was much less than that of CNF and DACNF. This may be due to the distinct mechanisms of different treatments. Ultrasonication involves utilizing acoustic cavitation to disrupt the hydrogen bonds between chitin molecules rather than to act on the intramolecular glycosidic bonds. In contrast, acid hydrolysis decomposes intramolecular structures, and therefore leads to CNCs with lower length than that of CNFs.

Paillet and Dufresne [34] prepared CNC by using β-chitin from squid pens. They obtained CNC with an average diameter of 10 nm and a length of 150 nm, respectively. The aspect ratio was calculated as 15, which corresponds with the result of our study. Moreover, CNCs made of crab-shell chitin had diameters ranging from 16 nm to 28 nm, lengths from 200 nm to 300 nm, and aspect ratios from 8.8 to 15 [28,29,33,35]. Using shrimp-shell chitin as raw material to prepare CNC, resulted in diameters ranging from 10 to 33 nm, lengths from 200 to 500 nm, and aspect ratios from 13.5 to 17 [36,37]. CNCs made of α-chitin revealed larger diameters and lengths together with a larger particle size. This may be due to that α-chitin possesses more extensive crystalline regions in structure than β-chitin, and it is therefore more difficult to hydrolyze α-chitin into small particles.

### 3.3. Functional Groups Identification and DD Determination of Chitins and its Nanomaterials

**Figure 3:**
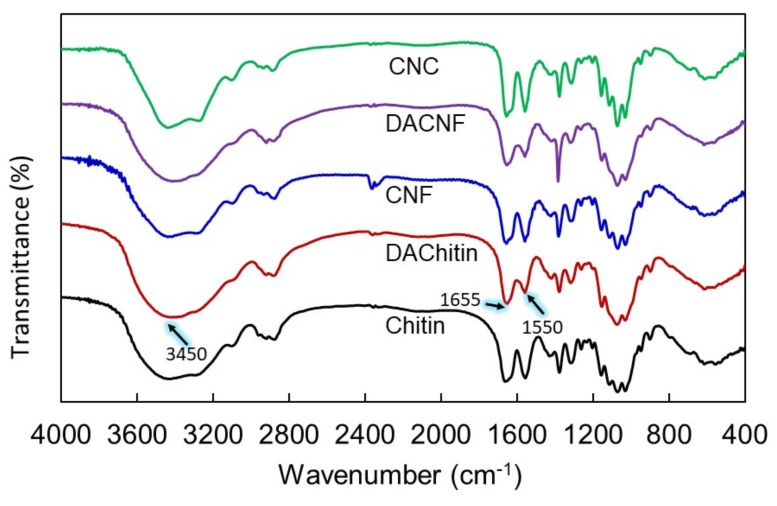
FTIR spectra of chitin, deacetylated chitin (DACTN), chitin nanofibers (CNF), deacetylated chitin nanofibers (DACNF), and chitin nanocrystals (CNC).

FTIR studies of chitin, deacetylated chitin, CNF, DACNF and CNC were performed to characterize the chemical structure and DD of those materials. The FTIR spectra of chitins and its nanomaterials are shown in Figure 3. In general, the FT-IR spectrum for chitin exhibits absorption peaks typically at 3450 cm^−1^ (OH stretching vibration), 1655 cm^−1^ (amide I, C=O vibrations) and 1550 cm^−1^ (amide II, a combination of CNH stretching and NH bending) [18,29]. In the FTIR spectrograms, weaker absorbance at amide I and amide II was observed in deacetylated chitin than in original chitin, indicating that the deacetylation have been conducted successfully. Meanwhile, chitin and deacetylated chitin revealed identical spectrum profiles at amide absorption peaks in the spectrograms, suggesting that the ultrasonication and acid hydrolysis provided a trivial effect on the chemical structures and DD of chitin.

The DD of samples were presented in Table 1. For raw materials, the DD of chitin and deacetylated chitin were 22.84% and 48.45%, respectively. Further fabrication process did not alter the chemical structure and DD of nanomaterials. The DD value of CNF, DACNF, and CNC were 22.89%, 53.36%, and 23.43%, respectively, and were not significantly different from their corresponding raw materials.

### 3.4. X-ray Diffraction

**Figure 4:**
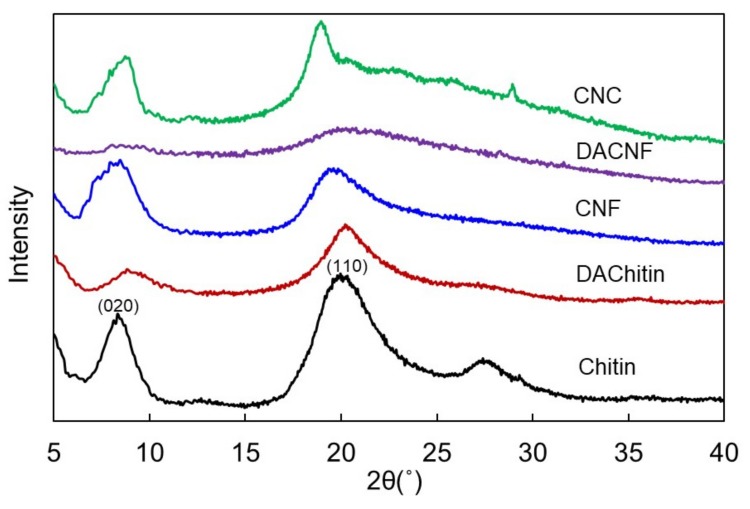
X-ray diffraction patterns of chitin, deacetylated chitin (DACTN), chitin nanofibers (CNF), deacetylated chitin nanofibers (DACNF), and chitin nanocrystals (CNC).

X-ray diffraction patterns of chitin and its nanomaterials are shown in Figure 4. There were two strong peaks in the diffractogram of chitin at 2θ at 8.5° and 19.7°, representing the crystalline reflections of chitin that were indexed as 020 and 110 from the lower angle for chitin [27,38]. Moreover, chitin showed another peak at 2θ at 27.5°, which corresponded to the planes of 013, with the result from Fiamingo et al. [39]. CNF and CNC possessed typical crystalline reflections of chitin in the present study, which were comparable to the results from CNF prepared by Lu et al. [18] and the CNC by Goodrich & Winter [36], respectively. In addition, it is worth mentioning that the peak at 2θ at 27.5° of chitin disappeared after being fabricated into its nanomaterials. This is the typical crystalline structure transition of β-chitin when being further fabricated [40]. Calculated crystalline indices were shown in Table 1. Results indicated that the crystalline indices of CNF and CNC were lower than its raw material, namely, β-chitin, implying that ultrasonication and acid hydrolysis processes moderately disrupted the crystallinity of materials.

Comparing to chitin, deacetylated chitin showed slightly shifted peaks in diffractogram in our results. Moreover, the peak intensity and crystalline index decreased with increasing DD of materials. These results were similar to those from previous reports, indicating that the deacetylation process with 33% NaOH at 90 °C for 4 h would disintegrate the crystalline arrangements of chitin [35]. Further ultrasonication process for micronizing deacetylated chitin into DACNF caused the reduction of peak intensities and crystalline indices as well. In the diffractogram, peak intensities of DACNF at 2θ at 8.5° and 20° were reduced. This may be due to the smaller amount of acetamide groups that deacetylated chitin possessed. Extensive hydrogen bonds may occur within the carbonyl oxygen of acetamide groups and chitin molecular chains [32]. Deacetylated chitins contain less acetamide groups, which may result in a more easily destructible crystalline region in physical structure.

### 3.5. Properties of Chitin Nanomaterials/NaCl Suspensions

#### 3.5.1. Zeta Potential

The available positive charges resulted from the presence of –NH3+ have been reported as important factors that influence the capability of chitin for enhancing saltiness perception [13]. The positive charge on the surface of a chitin can be represented by the zeta potential, so we examined the effects of concentration of various chitin nanomaterials on the zeta potential values.

**Figure 5:**
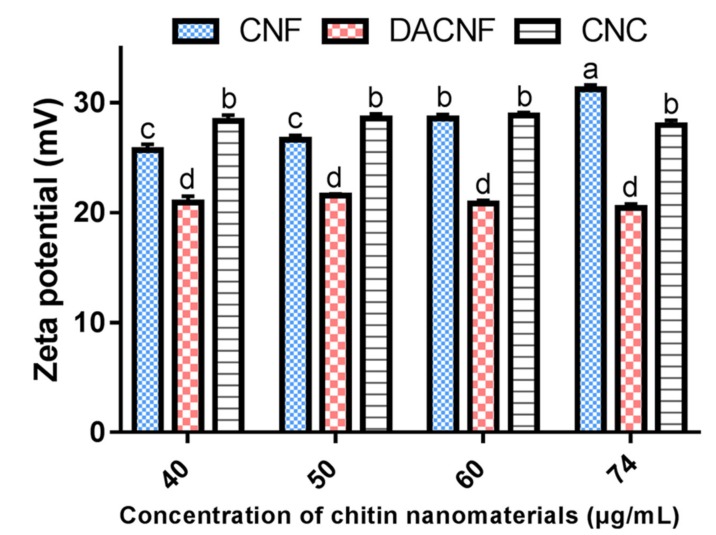
Zeta potentials of different concentrations of chitin nanofibers (CNF), deacetylated chitin nanofibers (DACNF), and chitin nanocrystals (CNC). ^a–d^ Value for each sample with different superscripts are significantly different (*p* < 0.05).

Zeta potential profiles of CNF, DACNF and CNC suspensions are shown in Figure 5. Obviously, the concentration of those nanomaterials has a negligible effect on zeta potential of its suspensions. The zeta potential values of CNF and CNC suspensions at a concentration of 74 μg/mL (ca. pH = 6) have positive charges of 30.08 mV and 28.58 mV respectively. Fan et al. [25] and Jiang et al. [13] presented similar results to our study. They prepared CNF from squid pens with zeta potential values at 40 mV and 31 mV at pH = 6, respectively. Pereira et al. [29] prepared CNC from crab shells with a zeta potential value around 30 mV, and their result was comparable to the present study as well.

The zeta potential of DACNF was 19.73 mV. Chang et al. [41] reported that chitosan molecules with a DD value of 95% would deprotonate at pH = 7, and were aggregated and possessed negative surface charge due to inter-/intramolecular hydrogen bond formation. Moreover, at pH = 6, the chitosan particles had a zeta potential of 16.23, which was comparable to that of chitin from our study. In the present study, although DACNF possessed more exposed amino groups than CNF and CNC, the zeta potential value of its suspension is still lower than that of CNF and CNC. This may be due to the presence of negative ions in the solution system. Free negative ions would be attracted by the protonated amino groups of chitin within diffuse layer, and then neutralize a portion of positive charged groups. The positive surface charge of DACNF particles was therefore reduced.

**Figure 6:**
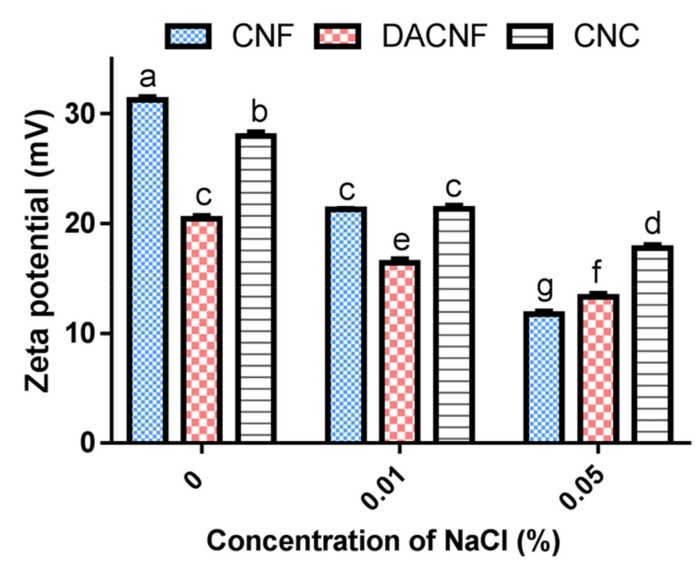
The effect of NaCl concentration on the zeta potentials of 74 μg/mL chitin nanofibers (CNF), deacetylated chitin nanofibers (DACNF), and chitin nanocrystals (CNC). ^a–g^ Value for each sample with different superscripts are significantly different (*p* < 0.05).

Figure 6 showed the zeta potential profiles of CNF, DACNF and CNC at a concentration of 74 μg/mL in various concentrations of NaCl solution. Apparently, the zeta potential values of chitin nanomaterials were reduced significantly along with increasing concentration of NaCl. This implies that chitin nanomaterials are capable of attracting free Cl^−^ ions to force the releasing of free Na^+^ ions and improve the saltiness perception in the food system [13].

#### 3.5.2. Sensory Evaluation

Table 3 listed the sensory evaluation results of chitin nanomaterials suspensions at a concentration of 80 μg/mL with a NaCl concentration of 0.3%. Results showed that CNF, CNC and DACNF groups tasted saltier compared with the control group (0.3% NaCl alone). Moreover, the saltiness perception of CNC and DACNF suspensions performed are higher than that of CNF suspension. Jiang et al. [13] reported that CNF at concentrations of 150 and 300 μg/mL promoted the saltiness perception of the NaCl solution. Chitin nanomaterials improved the saltiness perception owing to the protonation capability of its amino groups in faintly acidic environment, which would attract negative ions and enable more Na^+^ ions of salts to enter the receptor cells in taste buds via sodium ion channels, and improve the saltiness perception of foods [13,25].

Total quantities and surface areas of nanomaterials were listed in Table 2, which were calculated from the average lengths and diameters (assuming cylindrical shape, chitin density was 1.425 g cm^−3^) [42]. Results indicated that the quantities and surface areas of CNC and DACNF were larger than that of CNF, and improved their capabilities to adsorb negative ions in the system. Therefore, the addition of CNC and DACNF may promote much better saltiness perception than CNF. In addition, comparison of results from CNF and DACNF group revealed that the DD might play a crucial role in saltiness perception performed by corresponding chitin nanomaterials, which may be due a higher DD value represents a higher capability of chitin nanomaterials to attract more negative ions.

## 4. Conclusions

In the present study, chitin nanofibers with an aspect ratio greater than 100 were prepared from β-chitin from squid pens through ultrasonication treatment, while the rod-shaped nanocrystals with an aspect ratio of approximately 10 were fabricated by acid hydrolysis. For chitin nanofibers, higher DD value of raw material resulted in a reduced yield of product. In the preparation process of nanomaterials, crystalline arrangements of chitin molecules were disintegrated, and the crystalline indices reduced accordingly. However, the alterations on DD values were negligible. In addition, the zeta potential values of chitin nanomaterials suspensions were not affected by the concentration of nanomaterials, but would decrease with increasing concentrations of NaCl. On the other hand, chitin nanomaterials may become positively charged in suspension system with a pH value < 7 by the protonation of amino groups in chitin molecules. This characteristic enables chitin nanomaterials to adsorb negative ions, and increases the quantities of free Na^+^ ions in the system. The saltiness perceptions are therefore promoted. Moreover, positive relations were found between the saltiness perceptions and the quantity, surface area, and DD value of chitin nanomaterials. Consequently, we can conclude that these chitin nanomaterials are potent saltiness enhancers, and can be used for lowering the sodium levels of foods. This study investigated the physicochemical properties for chitin nanomaterials to improve saltiness perception, and helped to provide a promising starting point for the development of reduced-salt products such as cured foods, soups, and seasonings in food industry.

## Figures and Tables

**Table 1:** The yield, degree of deacetylation (DD) and crystalline index of chitin, deacetylated chitin (DAChitin), chitin nanofibers (CNF), deacetylated chitin nanofibers (DACNF), and chitin nanocrystals (CNC).

Sample	Method	Yield (%)	DD (%)	Crystalline Index (%)	
				CrI_110_	CrI_020_
**Chitin**			22.84 ± 1.97 ^a^	81.40	73.33
**CNF**	Ultrasonication	50.67 ± 4.93 ^b^	22.89 ± 1.05 ^a^	69.23	66.67
**CNC**	Acid hydrolysis	44.47 ± 3.44 ^b^	23.43 ± 1.41 ^a^	77.14	70.37
**DAChitin**			48.45 ± 0.91 ^b^	70.37	42.86
**DACNF**	Ultrasonication	19.00 ± 0.04 ^a^	53.36 ± 2.41 ^b^	57.14	34.07

All data were expressed as mean values ± S.D. (n = 3). ^a–b^ Value for each sample with different superscripts are significantly different (*p* < 0.05).

**Table 2:** The average diameter, length, aspect ratio, calculated number and surface area of chitin nanomaterials.

Item	CNF	DACNF	CNC
Average diameter (nm)	17.24 ± 2.02 ^a^	15.01 ± 3.57 ^c^	16.05 ± 1.10 ^b^
Average length (nm)	1725.05 ± 591.49 ^a^	1806.60 ± 496.71 ^a^	116.91 ± 2.06 ^b^
Aspect ratio	100.35	120.59	7.28
Volume (nm^3^)/CNF or CNC	4.038 × 10^5^	3.203 × 10^5^	2.365 × 10^4^
Surface area (nm^2^)/CNF or CNC	9.417 × 10^4^	8.571 × 10^4^	6.300 × 10^3^
Number/mL of CNF or CNC *	1.390 × 10^11^	1.753 × 10^11^	2.373 × 10^12^
Total surface area (nm^2^/mL)	1.309 × 10^16^	1.502 × 10^16^	1.495 × 10^16^

* The concentration of chitin nanomaterial was 80 μg/mL. The density of chitin was 1.425 g/cm^3^ refer to Rezaei et al. (2009). ^a–c^ Value for each sample with different superscripts are significantly different (*p* < 0.05).

**Table 3:** The salty sensory evaluation of chitin nanomaterials/0.3% NaCl solution.

Sample	0.3% NaCl	CNF + 0.3% NaCl	DACNF + 0.3% NaCl	CNC + 0.3% NaCl
**Saltiness**	3.17 ± 1.32 ^a^	4.07 ± 1.12 ^b^	4.83 ± 1.12 ^c^	4.83 ± 0.92 ^c^

All data were expressed as mean values ± S.D. (*n* = 30). ^a–c^ Value for each sample with different superscripts are significantly different (*p* < 0.05).
